# Predictors of delayed health seeking for febrile children: multi-level analysis of cross-sectional study data from southern Ethiopia

**DOI:** 10.3389/fpubh.2024.1417638

**Published:** 2024-09-09

**Authors:** Yilma Chisha, Tesfaye Feleke, Eshetu Andarge Zeleke, Zeleke Aschalew, Zeleke Girma Abate, Yosef Haile, Mulugeta Dalbo, Misganu Endriyas

**Affiliations:** ^1^School of Public Health, Arba Minch University, Arba Minch, Ethiopia; ^2^Flinders Health and Medical Research Institute, Flinders University, Adelaide, SA, Australia; ^3^Department of Public Health, Arba Minch College of Health Sciences, Arba Minch, Ethiopia; ^4^South Ethiopia Regional Public Health Institute, Jinka, Ethiopia

**Keywords:** delay treatment seeking, Gamo zone, Ethiopia, under five children, multi-level, prompt treatment seeking, fever

## Abstract

**Background:**

Febrile illnesses are commonly reported as a primary reason for seeking healthcare in sub-Saharan Africa. Timely diagnosis and getting prompt treatment within 24 h of fever onset is crucial to avert the risk of developing severe complications and death. Understanding factors contributing to delayed health seeking is important for public health interventions. Hence, this study aimed to assess individual- and contextual-level factors associated with the delay in seeking prompt treatment for children with fever.

**Method:**

A community-based cross-sectional study was conducted from September 2022 to June 2023 in Gamo zone, southern Ethiopia. Three districts were chosen, and then, from each district, six clusters or kebeles were chosen by simple random sampling. A total of 820 caregivers were randomly selected. A two-level mixed-effects logistic regression model was employed to identify factors associated with the delay in seeking prompt treatment. The associations were measured by an adjusted odds ratio (AOR), and statistical significance was declared at a 5% level of significance.

**Result:**

The prevalence of the delay in seeking prompt care was 47.8%. Factors contributing to the delay were caregivers who were aged 30 years and above [AOR 0.23, 95% confidence interval (CI): 0.10–0.52], caregivers who followed the Protestant religion (AOR 3.67, 95% CI: 2.08–6.48), caregivers unable to read and write (AOR 5.32, 95% CI: 6.80–11.70), merchant caregivers (AOR 6.63, 95% CI: 2.75–15.97), caregivers who were exposed to only one media source (AOR 9.3, 95% CI: 8.43–15.60), caregivers with the experience of child death (AOR 0.05, 95% CI: 0.01–0.22), and caregivers seeking permission from their partners to access healthcare (AOR 12.64, 95% CI: 6.98–22.89).

**Conclusion and recommendations:**

There was a high level of delay in seeking healthcare. Targeted community education through mass media, healthcare facilities, and community-level awareness campaigns should be strengthened to improve early treatment seeking and lessen the consequences of delayed treatment seeking.

## Introduction

Fever is a non-specific response to various infectious and non-infectious triggers. It is indeed a significant public health concern and a prevalent clinical symptom in children under 5 years of age who visit healthcare facilities (1, 2). Febrile illnesses are commonly reported as a primary reason for seeking healthcare in sub-Saharan Africa (SSA) (3, 4), and a substantial majority (over 80%) of pediatric consultations in resource-limited countries are attributed to febrile illnesses (5, 6).

In SSA, fever cases are often treated using a combination of modern and traditional medicines in community settings (7, 8). Fever can be associated with various childhood illnesses, including bacterial infections, parasitic infections, and upper or lower respiratory tract viral infections (9, 10). However, in areas where malaria is prevalent, fever is strongly indicative of a potential malaria infection and plays a significant role in influencing decisions regarding malaria treatment (11, 12). In health facilities across SSA, ~30–40% of all fever cases are attributed to malaria, with significant seasonal variations between rainy and dry seasons (13). Malaria is a major public health concern in Ethiopia, with ~75% of the country's landmass considered at risk of malaria (areas below 2,000 m altitude) and 68% of the population residing in malaria risk areas (14–17).

The World Health Organization (WHO) recommends confirming the presence of plasmodium species through testing before starting anti-malaria treatment (18). In addition, it is now evident that febrile children under the age of five should be investigated for other potential causes of fever as there are significant proportions of patients with fever caused by infections other than malaria (9, 19). It is important for caregivers of children to seek medical care within 24 h of the onset of fever to ensure timely diagnosis and appropriate management (9). If a child is not diagnosed early and does not receive prompt treatment, the child is at risk of developing severe complications and potential fatalities associated with malaria (20). Malaria is a significant global health issue, particularly in Africa. In 2015, it caused 429,000 estimated deaths worldwide, with over 75% of those deaths occurring in children under 5 years of age. Tragically, it is estimated that malaria claims the life of one child every 2 min (21, 22). In Africa alone, there are 300–500 million estimated cases of malaria each year, resulting in ~1 million malaria-related deaths.

More than 90% of these deaths occur in children under 5 years of age (23). In Ethiopia, malaria is the leading communicable disease, accounting for ~30% of the overall disability-adjusted life years lost (24). Annually, there are over five million new cases of malaria in Ethiopia (25, 26), making it the leading cause of outpatient visits, health facility admissions, and inpatient deaths for children under 5 years of age (27, 28). In 2016, an estimated US$ 2.7 billion was invested globally in malaria control and elimination efforts, with ~74% of the funding allocated to the WHO African Region. Despite these investments, malaria continues to be a significant burden for African countries (29). Studies have shown that many deaths related to febrile illnesses, including malaria, occur at home without patients receiving appropriate medical care, and when care is sought, it is often delayed (30–33). Prompt and effective treatment involves administering recommended medicines within 24 h of fever onset after confirming the root cause of the fever. Timely access to effective treatment, particularly with first-line artemisinin combination therapies (ACTs), is crucial in preventing the progression of the disease to the severe stage and reducing mortality, especially among children under 5 years of age. However, the proportion of febrile patients receiving prompt and effective treatment across SSA remains low (34–37).

It is true that populations in Ethiopia, including the population in Gamo zone, have been facing various diseases, both communicable and non-communicable. Among the communicable diseases, acute febrile illnesses, such as malaria, are of particular concern and have a significant impact on public health. In response to this, the Federal Ministry of Health (FMOH) has developed a national strategic plan for malaria prevention and control (38). This plan focuses on early diagnosis and prompt treatment, selective vector control, the use of insecticide-treated nets (ITNs) for mosquitoes, and environmental management. Despite these efforts, challenges still persist in effectively addressing the issue. Early diagnosis and prompt treatment are crucial in saving lives from acute febrile illnesses, including malaria. However, there is limited information on healthcare-seeking behavior among households of febrile children in Ethiopia, particularly in Gamo zone. This study aimed to fill the knowledge gap by examining individual- and contextual-level factors that influence prompt healthcare-seeking behavior of caregivers of febrile children under 5 years of age. By understanding these factors, we can improve strategies to encourage timely seeking of healthcare for febrile illnesses in Ethiopia.

## Methods and materials

### Study design, area, and period

A community-based cross-sectional survey was conducted in Gamo zone from September 2022 to June 2023. Gamo zone is in southern Ethiopia. Arbaminch town, the capital of Gamo zone, is situated ~505 km southwest of Addis Ababa, the capital of Ethiopia, and 134 km from Wolaita Soddo, the administrative and political center of South Ethiopia state. Administratively, the zone is subdivided into woredas (districts) and towns, which are again subdivided into kebeles. A kebele is the smallest administrative structure with an estimated population of 5,000. According to the 2007 census, the total population of Gamo zone was projected to be 1,341,901. The zone has one general hospital, five primary hospitals, 33 private clinics, and 56 public health centers according to the information gathered from officials of the health department. Malaria consistently ranks among the top 10 diseases for adults and the top five diseases for children every year, as reported by health officials.

### Population and eligibility criteria

The source population consisted of all caregivers living in Gamo zone who had children under the age of five diagnosed with fever before 1 month of the survey. The study population included households with children under the age of five who had fever within 1 month before the survey and met the inclusion criteria. These households were randomly selected from the selected kebele. Caregivers with children under the age of five who had fever within 1 month before the survey and who had been residing in Gamo zone for at least 6 months were included in the study. However, caregivers who were mentally handicapped or critically ill and unable to respond to the interview were excluded from the study.

### Sample size determination

The minimum sample size for this cross-sectional study was calculated using the one population proportion formula. The following assumptions were considered:

- The proportion of the delay in seeking prompt treatment among caregivers was 49.4% (39).
- The level of significance (α) was set at 5%.
- The standard normal curve or Z at a 95% confidence interval (CI; two-tailed) was 1.96.
- The margin of error (E) was 5%.
- The estimated power of the study was 80%.
- The formula used to calculate the sample size was
$$\begin{array}{r} n=\frac{{\left(Z_{\frac{\alpha }{2}}\right)}^{2}p(1-p)}{E^{2}}\\ n=\frac{1.96^{2}*0.494(1-0.494)}{0.05^{2}}=384 \end{array}$$
- To account for a 10% non-response rate, 38 caregivers were added to the sample size, resulting in a total of 422 caregivers. Finally, after multiplying the sample size by 2 (Design Effect), the total sample size required for the study was 844 caregivers.

### Sampling procedure

**Figure 1:**
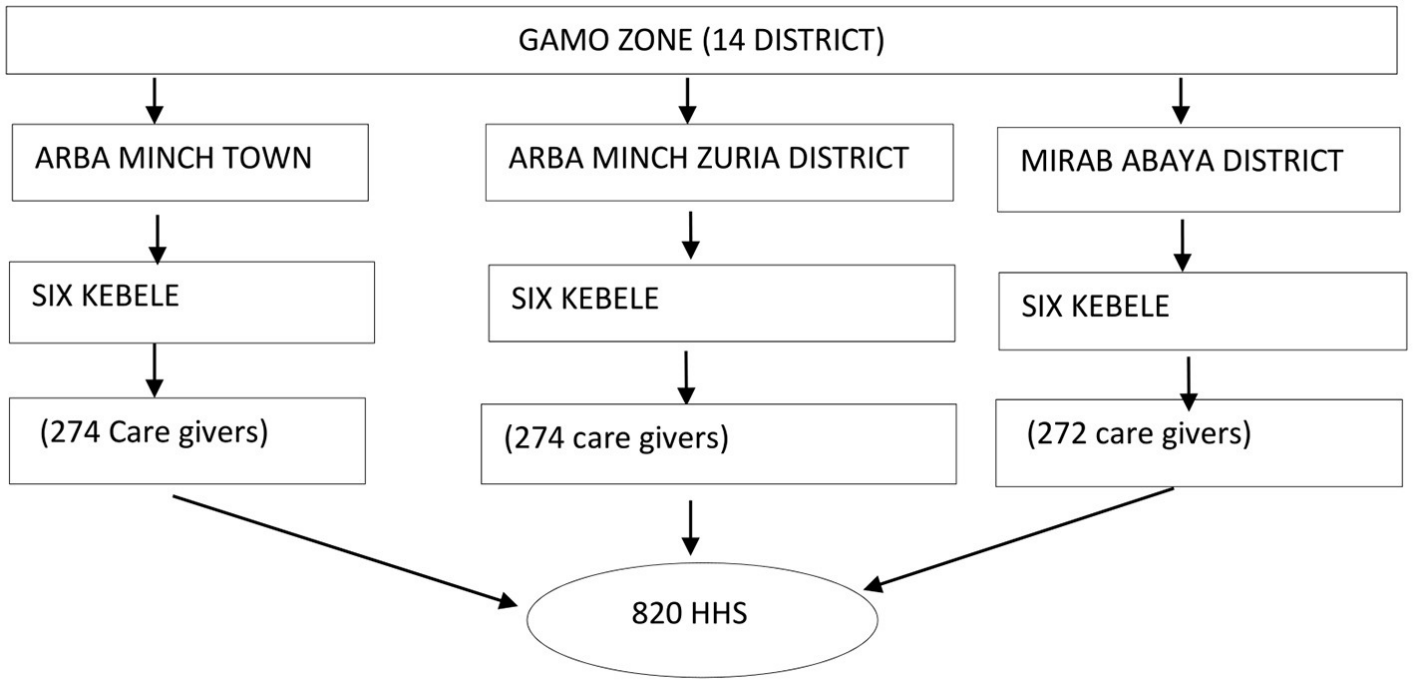
Schematic representation of the sampling procedure in Gamo zone, 2022/23.

The sampling procedure for this study involved selecting three out of 14 administrative districts through a simple random sampling method. From each of the three selected districts, six kebeles (clusters) were chosen again through the simple random sampling method, resulting in a total of 18 clusters for the study. Lists of households with children under the age of five who experienced fever within 1 month preceding the survey were obtained from each selected cluster and inputted into Microsoft Excel on Windows 10. Using this program, a random selection process was conducted to choose a total of 820. If there were two children under the age of five with fever in the household, the younger child was included (Figure 1).

### Data collection tool and procedure

The questionnaire used in the study was developed based on relevant literature from different parts of Ethiopia and Africa (46, 51, 52, 55) and adapted to the local context. It was translated from English to Amharic and then back to English by experts to ensure consistency. Nine diploma nurse data collectors and five degree-holding nurse supervisors were trained for 2 days by the investigators to ensure accurate data collection. They were trained on the purpose of the study, how to interview study subjects, and how to supervise the data collection process. A census was conducted, and we identified 1,356 households with children under the age of five who had experienced fever within 1 month before the survey. From this figure, 820 caregivers were randomly selected for the interview. Face-to-face interviews were conducted with the selected caregivers using interviewer-administered data collection techniques through a hand-held digital device using the Kobo collect toolbox data collection platform. In cases where the household members were not present during the initial data collection, a repeat visit was made. For households with multiple children under the age of five, the younger child was purposefully selected for data collection. In the case of households with twins, one child was selected using a lottery method. The data collection process spanned 15 consecutive days, and the data collection format included information on child-, household-, and community-level characteristics.

### Operational definitions

#### Multi-level modeling

Multi-level modeling refers to a modeling approach that is carried out for more than one level. This means that the data are organized in a hierarchical order, with units nested within each other.

#### Nesting

The clustering of units into a hierarchical order is referred to as nesting.

#### Fixed part of the model

The fixed part of a model, represented by Xβ, represents the average relationship between the predictor variable (X) and the outcome variable (Y). It is a component of the model that remains constant across all levels.

#### Delay in prompt treatment seeking

It refers to a situation where the caregiver takes children under the age of five to the healthcare facility after 24 h of fever onset.

#### Prompt and effective treatment

It refers to timely and appropriate treatment with recommended medicines within 24 h after the onset of fever.

#### Individual-level factors

In the context of multi-level modeling, individual-level factors refer to factors related to the child, household, and caregiver. These factors are specific to each individual within the hierarchical structure.

#### Contextual-level factors

These factors refer to community-related factors that may influence behavior or outcomes. They operate at a higher level than individual-level factors and can impact multiple individuals within the community.

#### Behavior

In this context, behavior refers to the response of an individual or group to an action, environment, or stimulus. It can encompass a wide range of actions or reactions exhibited by individuals or groups in different situations.

### Study variables

#### Dependent variable

The dependent variable for this research was the delay in healthcare seeking for febrile children under the age of 5 years. It was coded as “0” for non-delay and “1” for delay, with delay referring to seeking treatment after 24 h of fever onset.

#### Independent variables

To analyze factors influencing this delay, two sets of independent variables were considered due to the nested nature of the study. These sets included child- and household-level variables (individual-level variables) and contextual-level variables (community-level variables).

#### Child-level variables

The child-level variables included the age of the child in completed months, sex of the child, birth order of the child, and history of child death in the household.

#### Household-level variables

The household-level variables included the socio-demographic and other characteristics of caregivers, such as age and sex of the household leader, educational status, marital status, type of marriage, relationship of the caregiver with the child, occupation, monthly income, possession of health insurance, caregviers' media exposure and media asset availability at home, source of health information for the households, caregiver's perception of the capability of protecting his/her family from malaria, knowledge, perception, and practice toward malaria, religion, distance of the household to the nearest health facility, number of household members, number of children under the age of five at home, decision maker in the household for obtaining healthcare, and so on.

#### Community variables

**Figure 2:**
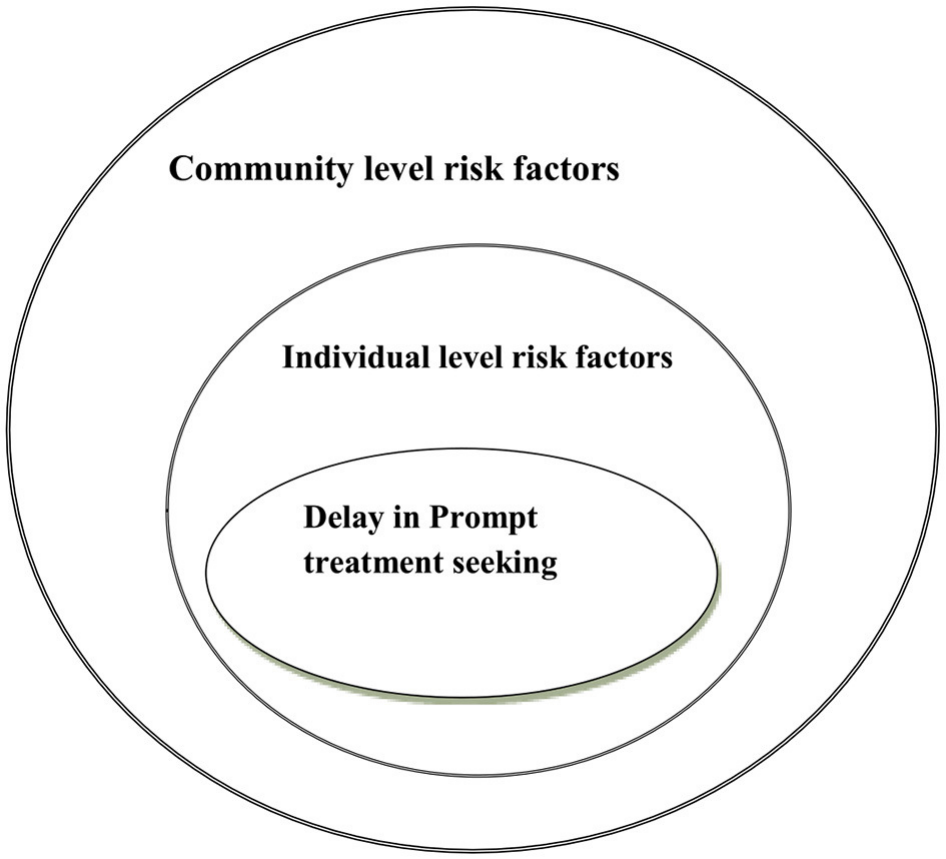
Conceptual framework of the individual- and contextual-level determinants of the delay in seeking prompt treatment for febrile children under the age of five, 2022.

The community-level variables included in this study were composed of place of residence and average score community-level educational status. Since the lower-level factors were nested on the higher-level factors (community), the framework was circular in shape (Figure 2).

### Data processing and management

The data collection for this study was conducted through a face-to-face interviewer-administered technique using a hand-held digital device and the Kobo collect toolbox data collection platform. Subsequently, the data were transferred to StataMP version 17 for data management and analysis purposes. Data cleaning procedures were performed to ensure the quality of the data. Furthermore, a test of normality, specifically the Shapiro–Wilk test, was conducted to identify and manage extreme values if they were present in the dataset. Coding, categorizing, labeling, and aggregating were also carried out as part of the data management process.

### Data quality assurance

Several measures were taken to ensure data quality. Data cleaning was performed to assess completeness, consistency, and extreme values. In addition, a 2-day training session was provided to both the data collectors and supervisors to ensure their understanding of the data collection process. A pre-test was conducted on 5% of caregivers (households) outside of the study clusters to validate the data collection tools (Eligo Wozeka). Based on the results of the pre-test, necessary corrections were made, and the questionnaire was further modified to improve its effectiveness. These steps were taken to enhance the accuracy and reliability of the collected data.

### Statistical analysis and presentation

Two-level (individual and community) data modeling was performed. Direct modeling of the clustered data was performed without sample weighting. This was possible because an equal number of samples were taken from each cluster. For continuous variables that followed a normal distribution, the mean (±SD) was used, while for continuous variables that did not follow a normal distribution, the median [interquartile range (IQR)] was used. Categorical predictors were presented as frequency and percentage.

In traditional or ordinary regression models, study subjects are assumed to be independent in relation to the outcome being studied. However, when data are structured in hierarchies, such as clusters, units within the same group are often not independent. The units within the same cluster tend to be more similar to each other in terms of the outcome of interest compared to the units from different clusters. This violation of the assumption of independence can lead to the underestimation of standard errors and an increased type I error rate (false-positive results). To address this issue, multi-level modeling was employed.

Multi-level modeling allows for simultaneous consideration of factors at both individual and community levels, providing a more robust understanding of the factors associated with the outcome variable. By accounting for the hierarchical structure of data, multi-level modeling helps mitigate the effects of violating the assumption of independence and improves the accuracy of results. The intra-community correlation (ICC) was calculated to measure the community effect and account for the clustered data nature, within-community and between-community variations. The ICC helps in determining the extent of similarity or correlation within each community. By considering the ICC, we could appropriately choose the method for determining the factors associated with prompt healthcare seeking among caregivers with children under the age of five. The ICC was approximated as follows:


$$\begin{array}{l} ICC=\;\frac{\sigma u^{2}}{\sigma u^{2}+\sigma e^{2}} \end{array}$$

where σu^2^ denotes community-level variance, and σe^2^ denotes individual-level variance that is fixed for log distribution to π^2^/3 or the value of 3.29. In this study, the value of the ICC being above zero indicates that the multi-level logistic regression model is considered better for predicting the outcome of interest compared to the traditional or standard binary logistic regression analysis model. A two-level mixed-effect logistic regression model was fitted to estimate the effects of explanatory variables and community-level random effects on the delay in treatment-seeking behavior. The first level represents the individual level, while the second level represents the community level. The model aimed to estimate both the independent (fixed) effects of the explanatory variables and the community-level random effects on the log probability of the delay in prompt treatment seeking. The specific formulation of the two-level multi-level model used to model the log probability of the delay in prompt treatment seeking is provided in the study, outlining the variables and their respective effects within the model. The log of the probability of the delay in prompt treatment seeking was modeled using the two-level multi-level model as follows:


$$\begin{array}{l} \log\left(\frac{\pi_{ij}}{1-\pi_{ij}}\right)=\beta_{0}+\beta_{1}X_{ij}+\beta_{2}Z_{ij}+u_{j} \end{array}$$

In the given context, i and j represent level 1 (individual) and level 2 (community or cluster) units, respectively. The variables X and Z refer to the individual- and community-level variables, respectively. The probability of the delay in treatment seeking by the ith caregiver in the jth community is represented by πij. The β's are fixed coefficients, indicating that for every one unit increase in X or Z (a set of predictor variables), there is a corresponding effect on the probability of the caregiver delaying prompt treatment seeking. The intercept, β0, represents the effect on the probability of the caregiver delaying treatment in the absence of any influence from predictors. The random effect Uj represents the effect of the community level or cluster level on the decision of the caregiver regarding treatment seeking. Statistical analysis was conducted to examine the relationship between the delay in treatment seeking and various predictors. The analysis involved building models, including a null model as a baseline for comparison. A median odds ratio (MOR) was calculated in the null model to measure the median value of the odds ratio to depict variations in the delay in treatment seeking between the randomly chosen households from the clusters that had high rates of delay and the households from the clusters that had low rates of delay. The proportional change in variance (PCV) was used to estimate the portion of the outcome variable explained by different models.

A stepwise backward elimination model-building procedure was employed, and information criteria [Akaike information criterion (AIC) and Bayesian information criterion (BIC)] were used for the model comparison and selection of the final parsimonious model. Interactions and cofounders were tested using the changes in the regression coefficients, with a cutoff point of 15–20% beta change (40). Multi-collinearity was assessed using the variance inflation factor (VIF) with a cutoff point of a mean VIF > 10 (41). The value of the mean VIF was 8.4. Based on the above, it was determined that there was no potential collinearity between covariates that violated our estimation. The classification of the data in the final model was evaluated using the receiver observed characteristics (ROC) curve, and ~92.4% of the data were correctly classified. The associations between the delay in treatment-seeking behavior and predictors were summarized using an adjusted odds ratio (AOR) and corresponding 95% confidence interval. Statistical significance was determined using the Wald chi-square test with a *p*-value threshold of 0.05.

### Ethical considerations

The study obtained ethical approval from the institutional research ethical review board of Arba Minch University. In addition, a letter of permission was obtained from the Gamo zone health department, selected districts, and kebeles. Before data collection, the participants were informed about the overall aim of the survey, and written informed consent was obtained from the head of the household or the children's caregivers. The participants were assigned identification numbers to ensure confidentiality. In cases febrile children were identified during the data collection, they were referred to the nearest health facility to receive appropriate healthcare.

## Results

### Socio-demographic characteristics of caregivers and children

Out of the initial 844 households with children under the age of five with fever that were expected to be included, 820 households were actually included in the study, resulting in a response rate of 97.2%. From the 820 caregivers, 409 (49.9%) sought care early, 374 (45.6%) delayed seeking care, and 37 (4.5%) did not seek care at all. Approximately two-thirds, 532 (67.9%), of the households had a male head. The majority of the caregivers 655 (83.7%) fell within the age group of 30 years and below. More than three-fourths, 607 (77.5%), of the study participants resided in rural areas. Out of the total study participants, 643 (82.1%) reported having only one child under the age of five at home (Table 1).

**Table 1:** Socio-demographic characteristics of the caregivers who delayed seeking care for febrile children in Gamo zone, Ethiopia 2022 (*N* = 783).

**Variable**	**Category**	**Delayed seeking car**		**Total**	**Percent**	***p*-value**
		**Yes**	**No**			
		***N* (%)**	***N* (%)**			
Sex of the household head	Male	246 (46.2)	286 (53.8)	532	67.9	0.214
	Female	128 (51.0)	123 (49.0)	251	32.1	
Age of the caregiver	≤30	321 (49.1)	334 (51.0)	655	83.7	0.115
	>30	53 (41.41)	75 (58.59)	128	16.4	
Marital status of the household head	Married	365 (50.3	360 (49.7)	725	92.6	<0.001
	Others	9 (15.5)	49 (84.5)	58	7.4	
If married, how many wives do you have (*N* = 725)	Monogamy	331 (49.7)	335 (57.6)	666	91.9	0.243
	Polygamy	34 (57.3)	25 (42.4)	59	8.14	
Residence	Urban	57 (32.4)	119 (67.6)	176	22.5	<0.001
	Rural	317 (52.2)	290 (47.8)	607	77.5	
Child and caregiver relationship	Father/Mother	308 (47.5)	341 (52.5)	649	82.9	0.705
	Others	66 (49.3)	68 (50.7)	134	17.1	
Family size	Less than six	259 (47.6)	285 (52.4)	544	69.5	0.896
	Six and above	115 (48.1)	124 (51.9)	239	30.5	
Number of children under the age of five at home	One	313 (48.7)	330 (51.3)	643	82.1	0.273
	Two and above	61 (43.6)	79 (56.4)	140	17.9	
Religion of the caregiver	Orthodox	102 (39.1)	159 (60.9)	261	33.3	0.001
	Protestant	272 (52.1)	250 (47.9)	522	66.7	
Educational status of the caregiver	Illiterate	78 (77.2)	23 (22.8)	101	12.9	<0.001
	Primary (1-18)	238 (63)	140 (37)	378	48.3	
	Secondary and above	58 (19.1)	246 (80.9)	304	38.8	
Occupational status of the caregiver	Farmer	113 (62.1)	69 (37.90	182	23.2	<0.000
	Merchant	34 (68.0)	16 (32.0)	50	6.4	
	Housewife	217 (59.0)	151 (41.0)	368	47.0	
	Govt. employee	2 (1.5)	130 (98.5)	132	16.9	
	Others	8 (15.7)	43 (84.3)	51	6.5	
Monthly household income	≤1,000 Birr	207 (72.1)	80 (27.9)	287	36.7	<0.000
	Above 1,000 Birr	167 (33.7)	329 (66.3)	496	63.3	
Media exposure of the caregiver	Not exposed	45 (100)	0 (0)	45	5.8	<0.000
	Exposed to one	172 (72.9)	64 (27.1)	236	30.1	
	Exposed to two	131 (39.9)	197 (60.1)	328	41.9	
	Exposed to three	26 (14.9)	148 (85.1)	174	22.2	
Sex of the child	Male	170 (47.5)	188 (52.5)	358	45.7	0.886
	Female	204 (48.0)	221 (52.0)	425	54.3	
Age of the child in months	0–11 months	60 (47.2)	67 (52.8)	127	16.2	0.716
	12–23 months	56 (45.9)	66 (54.1)	122	15.6	
	24–35 months	82 (48.2)	88 (51.8)	170	21.7	
	36–47 months	101 (45.5)	121 (54.5)	222	28.4	
	≥48 months	75 (52.8)	67 (47.2)	142	18.1	
History of child death	Yes	32 (71.1)	13 (28.9)	45	5.8	0.001
	No	342 (46.3)	396 (53.7)	738	94.2	
Birth interval of the child	First child	155 (49.1)	161 (50.9)	316	40.4	0.662
	Second child	114 (45.4)	137 (54.6)	251	32.1	
	Third and above	105 (48.6)	111 (51.4)	216	27.5	

According to the information provided, nearly half, 378 (48.3%), of the caregivers had an educational level of grades 1–8. In addition, ~101 (12.9%) of the caregivers reported that they had no education or literacy skills. In terms of occupation, nearly half, 368 (47.0%), of the caregivers identified themselves as housewives, followed by farmers. Furthermore, ~132 (16.9%) of the caregivers stated that they were government employees. Only 51 (6.5%) of the caregivers were engaged in other occupational identities, such as student, daily laborer, Bajaj driver, or fisherman. Regarding the monthly household income, 496 (63.3%) reported earning more than 1,000 birr per month (Table 1).

In addition, 328 (41.9%) caregivers mentioned having exposure to two media sources, namely television and radio, along with owning a mobile phone. On the other hand, only 45 (5.8%) caregivers reported not being exposed to any media sources. Regarding characteristics of the children, more than half, 425 (54.3%), were female. Approximately 222 (28.4%) children fell within the age category of 36–47 months. The median age of the children was 32.5 months, with an interquartile range (IQR) of 26 months (Table 1).

### Sources of information for malaria and its prevention

**Figure 3:**
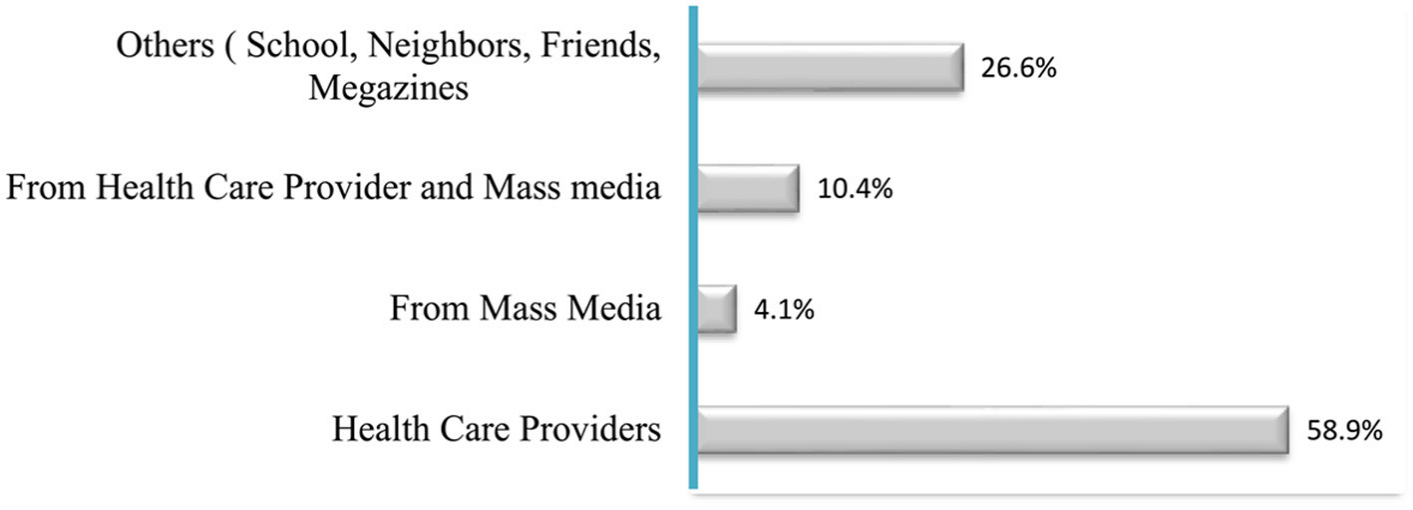
Percentage of the information sources for malaria and its prevention methods among the study participants, Gamo zone, 2022 (*N* = 820).

According to the information provided, all study participants in the study area were aware of the disease malaria. The primary source of information about malaria for the participants was healthcaregivers (Figure 3).

### Knowledge of malaria and its prevention methods

**Figure 4:**
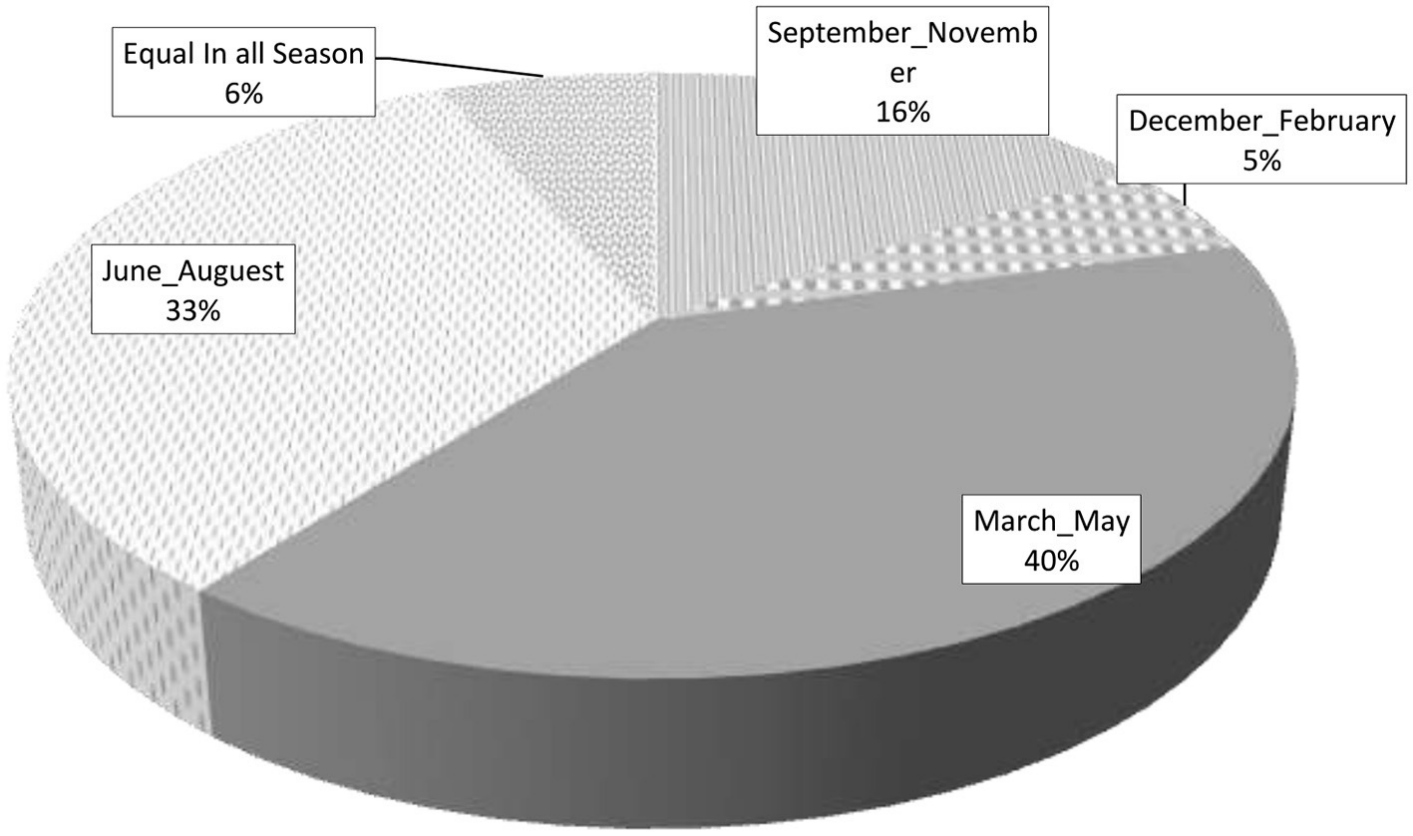
Percentage of the malaria peak season reported by the study participants, Gamo zone, 2022 (*N* = 820).

Based on the data provided, it was revealed that the majority (40%) of the respondents identified March to May as the peak season for the burden of malaria (Figure 4).

### Knowledge of caregivers about malaria and its prevention methods

It was evident that a significant portion of the respondents had knowledge about malaria and its prevention. The majority (77.6%) of the respondents recognized malaria as a public health problem in the study area. In addition, a high percentage of the respondents (80.6%) were aware of the use of modern medications for malaria, with a majority (84.6%) correctly identifying Coartem as the drug of choice. Furthermore, a large proportion of the participants (75.9%) were knowledgeable about the signs and symptoms of malaria and identified pregnant women and children under the age of five as the most at-risk population groups (73.8%). Moreover, a substantial number of the respondents (84.4%) were able to clearly report at least one of the major preventive methods for malaria. Nearly two-thirds of the participants (66.4%) knew about the major complications of untreated malaria, and only 58.5% of the participants correctly identified mosquito bites as the cause of malaria (Table 2). Based on the dichotomized scoring of the participants' knowledge out of 10 items, the mean score was calculated to be 6.95 ± 1.97. Out of the total participants, 424 (54.1%) scored at or above the mean, which was operationally defined as having good knowledge about malaria and its prevention. On the other hand, 359 (45.9%) of the participants scored below the mean, and they were categorized as having poor knowledge about this area (Table 2).

**Table 2:** Knowledge of the caregivers about malaria and its prevention methods and the delay in seeking care among the study participants in Gamo zone, South Ethiopia, 2022 (*N* = 783).

**Variable**	**Category**	**Delayed seeking car**		**Total**	**Percent**	***p*-value**
		**Yes**	**No**			
		***N* (%)**	***N* (%)**			
Is malaria a public health problem?	No	79 (45.1)	96 (54.9)	175	22.4	0.431
	Yes	295 (48.5)	313 (51.5)	608	77.6	
Is the cause of malaria a mosquito bite?	No	168 (51.7)	157 (48.3)	325	41.5	0.064
	Yes	206 (45.0)	252 (55.0)	458	58.5	
Malaria transmits from person to person.	No	145 (49.2)	150 (50.8)	295	37.7	0.546
	Yes	229 (46.9)	259 (53.1)	488	62.3	
Malaria transmits via mosquito bites (*N* = 488)	No	37 (75.5)	12 (24.5)	49	10.0	<0.001
	Yes	192 (43.7)	247 (56.3)	439	90.0	
Know the most at-risk population groups	No	95 (46.3)	110 (53.7)	205	26.2	0.635
	Yes	279 (48.3)	299 (51.7)	578	73.8	
Know the common signs and symptoms	No	86 (45.5)	103 (54.5)	189	24.1	0.475
	Yes	288 (48.5)	306 (51.5)	594	75.9	
Know the malaria confirmation method	No	134 (53.4)	117 (46.6)	251	32.1	0.031
	Yes	240 (45.1)	292 (54.9)	532	67.9	
Know the modern medications of malaria	No	101 (66.5)	51 (33.5)	152	19.4	<0.001
	Yes	273 (43.3)	358 (56.7)	631	80.6	
Know the major prevention methods of malaria	No	55 (48.3)	59 (51.7)	114	15.6	0.723
	Yes	287 (46.4)	331 (53.6)	618	84.4	
Know the complications ofuntreated malaria	No	96 (36.5)	167 (63.5)	263	33.6	<0.001
	Yes	278 (53.5)	242 (46.5)	520	66.4	
Overall knowledge of malaria	Poor	173 (48.2)	186 (51.8)	359	45.9	0.827
	Good	201 (47.4)	223 (52.6)	424	54.1	

### Healthcare-seeking behavior and other characteristics of the study participants

Out of 820 study participants, 783 (95.5%) took their febrile children to a healthcare facility and 37 (4.5%) gave homemade treatment. Among those who took their children to a healthcare facility, it was found that 374 (47.8%), 95% CI (44.3%, 51.3%), of the respondents in the study area experienced delay in seeking early treatment for their febrile children within 24 h. The majority of those who reported delay (56.7%) mentioned waiting for permission from their partner as a reason for the delay. In terms of healthcare costs, for more than half of the respondents, 424 (51.71%), the costs were covered by household pocket purchases, while for 396 respondents (48.29%), the healthcare costs were covered by the community-based health insurance (CBHI) scheme (Table 3).

**Table 3:** Healthcare seeking and other characteristics of the study participants, Gamo zone, 2022.

**Variables**	**Categories**	**Frequency**	**Percent**
If your child has fever, where do you take the child first?	Give homemade treatment	37	4.5
	Take to health facility	783	95.5
When do you take the child to the healthcare facility after fever onset? (*N* = 783)	Within 24 h	409	52.2
	After 24 h	374	47.8
Healthcare seeking for the febrile child	Seeking prompt treatment at the healthcare facility	409	49.88
	Delay in seeking treatment at the healthcare facility	374	45.61
	Give homemade treatment	37	4.51
Why do you delay in seeking treatment or not seek treatment at all? (*n* = 411)	Give homemade treatment	37	9.0
	Lack of transportation	2	0.49
	Lack of healthcare cost	86	20.92
	Waiting for permission from the partner	233	56.69
	Huge distance to the healthcare facility	13	3.16
	Lack of drugs at the healthcare facility	40	9.73
Are you a member of the CBHI scheme?	Yes	396	48.29
	No	424	51.71
Distance to the nearest facility	Mean 5.0 km ±(2.35 km)		
How long does it take on foot to the nearest healthcare facility	One hour or below	428	52.2
	Above 1 h	392	47.8

### Bivariable multi-level logistic regression analysis for candidate variable selection

Bivariable multi-level logistic regression analysis was conducted to select candidate variables for multivariable multi-level logistic regression analysis. The variables that met the preset criteria with a *p*-value of 0.25 and below were sex of the household head, age of the caregiver, marital status, type of marriage, residence, religion, educational status, average score community education, occupational status, monthly income, media exposure, history of child death, distance to the nearest health facility, and need for permission from the partner to obtain healthcare. These were identified as the candidate variables for the multivariable multi-level model (Table 4).

**Table 4:** Bivariable multi-level logistic regression analysis results, Gamo zone, 2022.

**Variable**	**Category**	**COR (95% CI)**	***p*-value**
Sex of the household head	Male	1	^*^0.21
	Female	1.21 (0.89, 1.63)	
Age of the caregiver	≤30	1	^*^0.12
	>30	0.74 (0.50, 1.08)	
Marital status of the household head	Married	1	^*^ <0.001
	Others	0.18 (0.09, 0.37)	
How many wives do you have?	Monogamy	1	^*^0.24
	Polygamy	1.37 (0.80, 2.36)	
Residence	Urban	1	^*^ <0.001
	Rural	2.28 (1.60, 3.25)	
Child and caregiver relationship	Father/Mother	1	0.71
	Others	1.07 0.74, 1.56)	
Family size	Less than six	1	0.89
	Six and above	1.02 (0.75, 1.38)	
Number of children under the age of five at home	One	1	0.27
	Two and above	0.81 (0.56, 1.17)	
Religion of the caregiver	Orthodox	1	^*^0.001
	Protestant	1.69 (1.25, 2.29)	
Educational status of the caregiver	Illiterate	14.38 (8.33, 24.83)	^*^ <0.001
	Primary (1-8)	7.21 (5.06, 10.27)	
	Secondary and above	1	
Occupation of the caregiver	Farmer	1	^*^ <0.001
	Merchant	1.30 (0.67, 2.52)	
	Housewife	0.88 (0.61, 1.26)	
	Govt. employee	0.01 (0.00, 0.04)	
	Others	0.11 (0.05, 0.26)	
Monthly income of the household of the child	≤1,000 Birr per month	5.09 (3.71, 7.00)	^*^ <0.001
	>1,000 Birr per month	1	
Media exposure of the caregiver	Not exposed		^*^ <0.001
	Exposed to one	15.30 (9.22, 25.37)	
	Exposed to two	3.79 (2.36, 6.07)	
	Exposed to three	1	
Sex of the child	Male	1	0.89
	Female	1.02 (0.77, 1.35)	
Age of the child in month	0–11 month	1	0.72
	12–23 month	0.95 (0.58, 1.56)	
	24–35 month	1.04 (0.66, 1.65)	
	36–47 month	0.93 (0.60, 1.44)	
	≥48 month	1.25 (0.77, 2.02)	
History of child death	Yes	1	^*^0.002
	No	0.35 (0.18, 0.68)	
Birth interval of the child	First child	1	0.662
	Second child	0.86 (0.62, 1.20)	
	Third and above	0.98 (0.70, 1.39)	
How is your healthcare cost covered?	By CBHI	1	0.264
	By pocket purchase	1.17 (0.89, 1.55)	
How long does it take to the nearest HCF on foot?	≤1 h	1	^*^ <0.001
	>1 h	1.98 (149, 2.63)	
Knowledge of malaria and its prevention	Poor knowledge	1.03 (0.78, 1.37)	0.827
	Good knowledge	1	
Need permission from the partner to obtain healthcare	Yes	9.4 (6.45, 13.70)	^*^ <0.001
	No	1	
Average score of community education	Poor education	0.79 (0.60, 1.05)	^*^0.105
	Good education	1	

Others in the child–caregiver relationship (sister, brother, stepmother, grandmother, and grandfather).

Others in the occupational status (student, daily laborer, Bajaj driver, and fisherman).

Others in the marital status (single, divorced, and widowed).

^*^Variables satisfied the p-value criteria of 0.25 or below.

1, Reference category.

### Factors associated with the delay in prompt treatment seeking for febrile children

The intraclass correlation coefficient (ICC) in the null model was found to be 6%, indicating that 6% of the variability in the delay in prompt treatment seeking for febrile children under the age of five was attributed to differences between the clusters or unobserved factors at the community level. The Akaike information criterion (AIC) in model 4 was the smallest, which was 551.27, making it the best-fitted model for the data. Therefore, all the interpretations and reports were based on model 4. Furthermore, the median odds ratio (MOR) in the null model was >1, suggesting variations in the delay in prompt treatment seeking between the study clusters in the zone. When we randomly selected two caregivers from the clusters with a high rate and low rate of delay, the caregivers from the cluster with a high rate of delay were 52% more likely to access treatment after 24 h for febrile children compared to their counterparts (MOD = 1.52). The high level of variance in the delay in seeking prompt treatment for febrile children was contributed by caregiver-related factors (variance 22.7, MOR 1.58). In addition, the proportional change in variance (PCV) value in the fourth model indicated that almost 39.6% of the variability in the delay in prompt treatment seeking was explained or determined by both individual-level and community-level predictors. This highlights the importance of considering both individual- and community-level factors in understanding and addressing delays in seeking prompt treatment (Table 5).

**Table 5:** Model summary of all models used in the analysis.

**Parameter**	**Null (model I)**	**Model II (individual level)**	**Model III (community level)**	**Model IV (mixed model)**
ICC	(6%)	(6.5%)	(3.4%)	(5.5%)
Variance	(19.4%)	(22.7%)	11.6%	19.2%
Proportional change in variance	Reference	32.5%	11.5%	39.6%
MOR (median odds ratio)	1.52	1.58	0.87	1.50
AIC	1,087.90	558.57	1,066.80	551.27
BIC	1,097.23	626.34	1,080.79	618.07

Out of 14 variables that met the preset *p*-value criteria of 0.25 to be considered candidate variables for the multivariable multi-level logistic regression analysis, half of them (50%) were identified as predictors of the delay in seeking prompt treatment in the multivariable analysis. These predictors included the following: age of the caregiver, educational status of the caregiver, occupation of the caregiver, religious preference of the caregiver, media exposure status of the caregiver, history of child death at the home, and need for permission to access healthcare services from the partner. These variables were found to have a significant impact on the delay in seeking prompt treatment. This information can help in understanding the factors influencing delays in accessing healthcare services and can guide interventions to improve timely treatment seeking (Table 6).

**Table 6:** Multivariable multi-level logistic regression analysis of the factors associated with the delay in seeking prompt treatment among the caregivers with febrile children, Gamo zone, 2022.

**Variables**	**Categories**	**AOR [95% CI]**		
		**Model: II (individual level)**	**Model: III (community level)**	**Model: IV (mixed model)**
Sex of the caregivers	Male	1		1
	Female	0.64 [0.34, 1.20]		0.64 [0.35–1.19]
Age group of the caregivers	≤30	1		1
	>30	0.23 [0.10, 0.54]		^*^0.23 [0.10–0.52]
Marital status of the caregivers	Married	1		1
	Others	0.97 [0.74, 1.27]		0.86 [0.77–1.32]
Type of marriage	Monogamy	1		1
	Polygamy	0.67 [0.26, 1.69]		0.74 [0.29–1.89]
Residence	Urban		1	1
	Rural		2.57 [1.70–3.90]	1.65 [0.81–3.36]
Religion of the caregivers	Orthodox	1		1
	Protestant	2.76 [1.52, 5.02]		^*^3.67 [2.08–6.48]
Educational status of the caregivers	Illiterate	5.80[3.84, 8.56]		^*^5.32 [6.80–11.20]
	Primary (1-8)	5.25 [2.91, 9.45]		^*^4.96 [2.82–8.71]
	Secondary and above	1		1
Occupation of the caregivers	Farmer	1		1
	Merchant	6.09 [2.39–15.55]		^*^6.63 [2.75–15.97]
	Housewife	3.41 [1.62–7.21]		^*^3.00 [1.59–5.68]
	Govt. employee	0.07 [0.01–0.34]		^*^0.05 [0.01–0.26]
	Others	0.32 [0.16–1.65]		0.54 [0.22–1.29]
Monthly income of the households	≤1,000 birr/month	1.51 [0.85–2.70]		1.45 [0.81–2.58]
	>1,000 birr/month	1		1
Media exposure status of the households	Not exposed	1.77 [0.67–2.33]		2.31 [0.58–3.03]
	Exposed to one	8.31 [5.56–11.89]		^*^9.30 [11.43–15.60]
	Exposed to two	7.81 [3.10–19.66]		^*^6.76 [2.82–16.20]
	Exposed to three	1		1
History of child death	Yes	0.05 [0.11–0.24]		^*^0.05 [0.01–0.22]
	No	1		1
Distance to the nearest healthcare facility on foot	≤1 h	1		1
	>1 h	1.73 [0.99–3.04]		1.65 [0.97–2.83]
Need permission from the partner to obtain healthcare services	Yes	13.11 [7.04–24.40]		^*^12.64 [6.98–22.89]
	No	1		1
Average score of community-level education	Poor education		1.20 [0.86–1.68]	0.91 [0.43–1.91]
	Good education		1	1

^*^Significantly associated variables.

According to the multivariable multi-level analysis, caregivers aged 30 years and above were 77% (AOR 95% CI: 0.23 [0.10–0.52]) less likely to delay in seeking prompt treatment for febrile children compared to the age of the caregiver being 30 years or younger. In addition, the caregivers who followed the Protestant religion were found to be 3.67 (AOR 95% CI: 3.67 [2.08–6.48]) times more likely to delay in seeking prompt treatment for febrile children compared to the caregivers who followed the Orthodox religion. The educational status of the caregivers was found to be statistically and significantly associated with the delay in seeking prompt treatment for febrile children. Specifically, the caregivers who were unable to read and write or illiterate were 5.32 (AOR 95% CI: 5.32 [6.80–11.70]) times more likely to delay in seeking prompt treatment compared to the caregivers who had a high school education or above. Similarly, the caregivers who had completed primary-level education (Grades 1–8) were 4.96 (AOR 95% CI: 4.96 [2.82–8.71]) were almost five times more likely to delay in seeking prompt treatment compared to the caregivers with a high school education or above. The occupation of the caregivers was found to have a significant impact on the delay in seeking prompt treatment for febrile children.

Merchant caregivers were 6.63 (AOR: 95% CI: 6.63 [2.75–15.97]) times more likely to delay in seeking treatment within 24 h compared to farmer caregivers. Similarly, the caregivers who were housewives were three times (AOR 95% CI: 3.00 [1.59–5.68]) more likely to delay in seeking prompt treatment compared to the caregivers who were farmers. On the other hand, government employees exhibited better prompt treatment-seeking behavior compared to farmers. The odds of the delay in seeking treatment within 24 h for febrile children were 95% less likely (AOR 95% CI: 0.05 [0.01–0.26]) for the caregivers who were government employees compared to the caregivers who were farmers. The number of media sources to which the caregivers were exposed was found to impact the delay in seeking prompt treatment for febrile children. The caregivers who were exposed to only one media source were 9.3 times (AOR 95% CI: 9.3 [8.43–15.60]) more likely to delay seeking treatment within 24 h compared to the caregivers who were exposed to three or more media sources. Similarly, the caregivers who were exposed to two media sources were 6.76 times (AOR 95% CI: 6.76 [2.82–16.20]) more likely to delay in seeking prompt treatment within 24 h compared to the caregivers who were exposed to three or more media sources. The caregivers who had previously experienced a child death were found to be 95% (AOR 95% CI: 0.05 [0.01–0.22]) less likely to delay in seeking prompt treatment compared to the caregivers with no history of previous child death. Seeking permission from the partner before accessing healthcare services for a child with fever was found to be significantly associated with the delay in seeking treatment within 24 h. The caregivers who required permission from their partners were found to be over 10 times more likely to experience delay in seeking prompt treatment for a child with fever compared to their counterparts (AOR 95% CI: 12.64 [6.98–22.89]; Table 6).

## Discussion

The current study highlighted that 47.8% (95% CI: 44.3–51.3%) of the caregivers delayed seeking early treatment for febrile children, indicating that only 52.2% sought prompt treatment within 24 h. This finding aligns with a similar multicounty study in SSA, where Kenya reported 50.6% prompt treatment and 49.4% delay (42). However, the percentage was lower than that of other countries, such as Angola, 57.8%; Benin, 77.8%; Ghana, 55.2%; Liberia, 61.3%; Malawi, 73.3%; Mozambique, 74.4%; Rwanda, 70.8%; Senegal, 73.0%; Tanzania, 68.9%; Uganda, 65.4%; Zambia, 53.4%; and Zanzibar, 87.2% (42–46). The variation in the results could be attributed to the zonal focus of the current study compared to the broader national scope of the previous studies. In addition, social desirability bias might have influenced responses, leading to potential over-reporting of prompt treatment-seeking behavior. This information underscores the importance of addressing delays in seeking healthcare for febrile children to improve overall health outcomes.

The study indicated that the caregivers aged over 30 years were 77% less likely to delay seeking prompt treatment for febrile children compared to the caregivers aged 30 years or younger. This finding is consistent with the findings of previous studies (47, 48), which suggested that older caregivers are more inclined to seek appropriate healthcare for their children. This might be due to older caregivers often having more experience in providing adequate care and knowing when to seek treatment for sick children. In addition, individuals over 30 years could be financially more stable, which reduces the likelihood of delaying treatment due to economic constraints. However, other studies conducted in the Rwimi town of Uganda (49) and the Mandura district in southwest Ethiopia (46) revealed that caregivers aged 30 years or younger were significantly more likely to seek early treatment within 24 h compared to caregivers over the age of 30 years. This difference could be attributed to younger caregivers potentially having fewer household responsibilities and more time and energy to seek treatment for their children, including being able to walk long distances if needed. The roles and responsibilities at home increase when the age of the caregiver increases. Further research through prospective cohort studies could help identify the real-life determinants behind these inconsistencies.

This research indicated that the caregivers who were Protestant followers were 3.67 times more likely to delay seeking prompt treatment for febrile children compared to those of the Orthodox faith. This aligns with a similar study in Kigali, Rwanda (50), which suggested that gospel believer caregivers might first turn to special prayers offered by the ministers of God before seeking medical care. If the child's illness or fever does not improve after these prayers, they would then seek medical treatment at healthcare facilities. These cultural and religious practices may influence the timing of seeking medical help. The educational status of the caregivers played a statistically significant role in the prompt treatment seeking for febrile children. Those who were illiterate or had completed only primary education were more likely to delay seeking care compared to those with a high school education and above. This finding is consistent with studies from Rwanda (49), Tanzania (51), and Shashogo District, Hadiya zone (52). This may be because caregivers with secondary-level education are more likely to seek treatment early within 24 h, possibly due to increased awareness and knowledge, which leads to timely recognition of symptoms and complications of malaria. Education can indeed empower caregivers to act promptly in seeking care for febrile children (53, 54). The likelihood of the delay in seeking prompt treatment for febrile children was significantly lower among the caregivers who were government employees compared to the caregivers with other occupations. It aligns with a study conducted in Gambella town (55). This better prompt treatment-seeking behavior of the government employees may be attributed to the fact that government employees generally have higher educational levels and better access to mass media, and some may even have backgrounds in healthcare. These factors likely contribute to their ability to recognize and respond promptly to signs of illness in children.

The study findings suggested that the caregivers who were exposed to multiple (three and above) media sources were more likely to seek prompt treatment within 24 h compared to the caregivers who were exposed to only one or two media sources. This result is congruent with a study finding in Tanzania (51). Caregivers exposed to more than one type of mass media source have higher odds of seeking appropriate care when they have febrile children. This could be attributed to the messages on malaria prevention and management delivered through mass media, emphasizing the importance of using insecticide-treated nets, getting tested for malaria before taking antimalarial, and understanding that not all fevers are malaria-related. Mass media have been shown to have a similar positive impact on healthcare facility deliveries and contraceptive use (56).

It is interesting to note that the caregivers with a history of child death at home were significantly less likely to delay seeking prompt treatment compared to the caregivers with no history of child death at home. This finding is consistent with similar studies conducted in Shashogo District, Hadiya zone (52), Gambella Town (55), and Jimma zone (31). It suggests that past experiences of child death may influence caregivers' decision to seek treatment more promptly in the future. This insight could potentially help improve healthcare practices for children in need of timely medical attention.

It is important for caregivers to have the autonomy to seek prompt treatment for their child's health needs, especially in urgent situations, such as when a child has fever. When caregivers need permission from their partners to access healthcare services, it can lead to delay in seeking treatment within the 24-h window. This delay can have serious consequences on the child's health. Caregivers should be empowered to make timely decisions regarding their child's health without unnecessary barriers.

The study's usage of multi-level modeling analysis to identify determinants of delay in seeking timely treatment for febrile children is commendable. However, it is important to acknowledge the potential biases that may have influenced the results, such as recall bias and social desirability bias. As a policy and public health implication, this study recommends implementing cost-effective technology-based health education and behavior change communication interventions at the community level. Policymakers, officials, and professionals can work together to reduce the prevalence of delay in seeking healthcare and improve health outcomes for children by leveraging cost-effective technologies and targeted communication strategies.

## Conclusion

Almost half of the caregivers delayed seeking medical care for febrile children in the study area. The caregivers who were aged 30 years or younger, caregivers who followed the Protestant religion, caregivers who had lower educational status, caregivers who were merchants or housewives, caregivers who had lower media exposure, caregivers who had no history of child death, and caregivers who needed permission from their partner to access healthcare were more likely to delay seeking healthcare. Targeted intervention should be implemented for caregivers with these identified risk factors to address the delayed care-seeking behavior. Health education and awareness campaigns through mass media, healthcare facilities, and community initiatives can help emphasize the importance of seeking early treatment and the consequences of delaying care for sick children and family members.

## Data Availability

The original contributions presented in the study are included in the article/Supplementary material. Further inquiries can be directed to the corresponding author.
