# Supplementary material for: Predictors of delayed health seeking for febrile children: multi-level analysis of cross-sectional study data from southern Ethiopia
Source: Front Public Health. 2024 Sep 9;12:1417638. doi: 10.3389/fpubh.2024.1417638 (PMC11423542; doi:10.3389/fpubh.2024.1417638)
Supplement: Supplementary file 1 [file Data_Sheet_1.docx]

**Annex: supplementary document**

As the curve shows, about 92.4% of the data was correctly classified in the final model.

Mean VIF=8.43, hence it indicates no potentially significant multi-collinearity
